# Peripheral Nerve Blockade

**DOI:** 10.1155/2011/973239

**Published:** 2011-07-28

**Authors:** Attila Bondar, Didier Morau, Sridhar Kollipara, Gabriella Iohom

**Affiliations:** ^1^Department of Anaesthesiology and Intensive Care Medicine, Semmelweis University, Kutvolgyi ut 4, 1125 Budapest, Hungary; ^2^Department of Anaesthesia and Intensive Care Medicine, Lapeyronie Hospital, Montpellier, France; ^3^Department of Anesthesiology and Pain Medicine, Western Memorial Regional Hospital, 1 Brookfield Avenue, Corner Brook, NL, Canada A2H 6J7; ^4^Department of Anaesthesia and Intensive Care Medicine, Cork University Hospital, Cork, Ireland

Regional anesthesia has gained significant popularity over the last few decades due to improved efficacy and patient safety. An increasing number of anesthesiologists perform peripheral nerve block (PNB) techniques in their daily clinical practice making the current special issue both timely and practical. 

Undoubtedly, PNB is the cornerstone of multimodal analgesia. Effective PNB removes the need for general anaesthesia (and its associated risks and sequelae). Aggressive perioperative analgesia prevents persistent postsurgical pain and may lead to improved clinical outcome. Patients can be discharged from the operating room directly to the ward, bypassing the postanesthesia care unit. Effective pain relief facilitates early physiotherapy, functional recovery, early ambulation, and discharge from the hospital, thus attracting serious cost savings. 

Ultrasound guidance has emerged as the “gold standard” in regional anesthesia or it is very close to it! In spite of all visual clues though, ultrasound guidance is incapable of preventing nerve injury or inadvertent intravascular injection; therefore, the usual precautions apply. Current trends in ultrasound-guided regional anesthesia point towards local anesthetic volume reductions. We have learned from recent research that a great deal of nerve stimulation guided blocks were actually intraneural, leading to the known minor, transient, or no neurological symptoms. More than ever before, formal structured training is of paramount importance for the safe practice of regional anesthesia. American Society of Regional Anesthesia and European Society of Regional Anaesthesia Joint Committee guidelines for training in ultrasound-guided regional anesthesia were produced in response to the “dramatic” increase in the use of ultrasound-guided PNBs in recent years.

The current special issue is a representative snapshot of our current understanding of PNB. We believe that the articles are informative and most of all thought provoking.

In “*Perioperative nerve blockade: clues from the bench*” M. R. Suter et al. tease out the effects of nerve blockade on pain-related behaviour and central changes after peripheral tissue injury in animal models. They propose mechanisms for the mitigated results of clinical studies on chronic postoperative pain.

“*Histological consequences of needle-nerve contact following nerve stimulation in a pig model*” by T. Steinfeldt et al. explores an interesting concept, that is, that high threshold current (1.0 mA) is associated with a reduced likelihood of direct needle-nerve contact compared with low threshold current (0.2 mA), thereby reducing the potential for needle-related nerve injury in an open pig brachial plexus model. The authors demonstrated that (i) low current threshold is likely to result in a greater incidence of needle-nerve contact, (ii) needle-nerve contact may result in aseptic neuroinflammation, but (iii) this inflammatory response, although greater following application of low (0.2 mA) compared to high (1 mA) current thresholds, is independent of the presence or absence of current.

Accepting such high current thresholds in clinical practice, in order to avoid needle-nerve contact may result in higher failure rates. The risk-benefit ratio of PNB may be improved when combining blind electrostimulation with ultrasound guidance. 

“*Neural blockade anesthesia of the mandibular nerve and its terminal branches: rationale for different anesthetic techniques including their advantages and disadvantages*” by J. Khoury and G. Townsend may seem an outlier at first sight. Although not part of the anesthesiologists' routine armamentarium, mandibular nerve blockade serves as an example of correlating anatomy with different techniques in a clear, unambiguous manner. Inquisitive clinicians will welcome both the approach and the content of this selective review. 

The article entitled “*Ultrasound-guided regional anesthesia for procedures of the upper extremity*” by F. Mirza and A. R. Brown describes various approaches to the brachial plexus in terms of their indications and advantages/disadvantages pointing out the value of ultrasound guidance in their performance. 

“*Axillary brachial plexus block*” by A. R. Satapathy and D. M. Coventry makes for a comprehensive description of the safest upper limb block, that is, ultrasound-guided axillary approach to brachial plexus blockade including clinical pearls and tricks of the trade.

In “*Ultrasound guidance for deep peripheral nerve blocks: a brief review*,” A. Wadhwa et al. review the role of ultrasonography in carrying out deep nerve or plexus blockade, such as infraclavicular, lumbar plexus, and sciatic nerve blocks. The authors highlight technical problems associated with scanning and needling deep structures. They caution that, based on the current literature, ultrasound does not replace experience and knowledge of relevant anatomy, especially for visualising deep structures. 

In “*The psoas compartment block for hip surgery the past, present, and future*” M. A. de Leeuw et al. provide an overview of the history, clinical efficacy, and risk profile of the psoas compartment block for hip surgery. While clearly, the block is a valuable adjunct to analgesia following hip surgery, further research is required to optimize the technique for usage as a sole anesthetic technique.

We hope the current selection of peer-reviewed articles will make both an interesting read and a useful reference material. For this, we thank the authors and the reviewers. We also wish to express our gratitude to the extremely supportive editorial staff at Hindawi Publishing Corporation, Ms. Amira Tyseer.

*Attila Bondar* *Didier Morau* *Sridhar Kollipara* *Gabriella Iohom*

